# Executive Summary to the Royal Society report “COVID-19: examining the effectiveness of non-pharmaceutical interventions”

**DOI:** 10.1098/rsta.2023.0211

**Published:** 2023-10-09

**Authors:** Professor Sir Mark Walport

**Affiliations:** Faculty of Medicine, Imperial College London and the Royal Society

**Keywords:** COVID-19, non-pharmaceutical interventions

## 1. Introduction

This issue of the Royal Society’s Philosophical Transactions A presents six evidence reviews developed in support of a Royal Society report entitled “COVID-19: examining the effectiveness of Non-Pharmaceutical Interventions” [1]. The conclusions of these reviews together with other input to the report are presented in this introduction to this themed issue in the form of the executive summary for that report. Non-pharmaceutical interventions (NPIs) were a set of measures (described in box 1) aimed at reducing the person-to-person transmission of severe acute respiratory syndrome coronavirus 2 (SARS-CoV-2), the virus that caused the pandemic.

Six groups of researchers were commissioned to assemble evidence reviews for this report, examining the effectiveness of a range of NPIs that were applied with the aim of reducing the transmission of SARS-CoV-2. Researchers were tasked with documenting what has been learnt, identifying gaps in knowledge and considering how these might be filled in the future. This report summarises these evidence reviews and interprets them alongside national case studies. It pays particular attention to the context and the constraints on the types of research that could be and were performed during the pandemic.

The report is non-judgemental on the timing and manner in which NPIs were applied in different regions and countries around the world. It focuses on understanding the impact of NPIs on SARS-CoV-2 transmission and makes no assessment of the economic or other societal impacts of the different NPIs. Assessing these other impacts are important tasks for the many different COVID-19 inquiries that are underway around the world.

Box 1. **What are non-pharmaceutical interventions (NPIs)?**

NPIs include any measure that is implemented during an infectious disease outbreak to attempt to reduce transmission that is not a vaccine or drug. NPIs can be behavioural, social, physical, or regulatory in nature. Their uptake and use can be encouraged through a variety of approaches, escalating from advice and guidance through to regulation. NPIs are therefore the first line of defence in the effort to contain outbreaks and to limit the impacts on affected populations before biological interventions become available. They have also been used alongside vaccines and drugs, especially where these interventions fail to prevent transmission. The precise ways in which NPIs were implemented during the COVID-19 pandemic varied between different countries and contexts.

The programme of work covered six broad categories of NPIs and the evidence available concerning their effectiveness at reducing transmission of SARS-CoV-2. The six categories are as follows:

**Masks and face coverings**

Masks act as barriers to virus particles in air being inhaled and/or exhaled through the nose or mouth. Virus-carrying droplets (larger, heavier particles) or aerosols (smaller, lighter particles) [2] captured on the inside or outside of the mask can no longer spread via the air. The materials and features of masks affect the size of the particles that are filtered out, and their resulting effectiveness. How well the mask fits the face of the wearer is also key. N95 masks (also known as respirators), when worn correctly, are highly effective barriers.

**Social distancing and ‘lockdowns’**

Respiratory diseases are transmitted by infectious material carried by exhalations (eg breathing, talking, coughing or sneezing) from one individual to another. Increasing physical distance between individuals can reduce the amount of infectious material being carried to others in droplets and aerosols, although aerosols typically transmit over longer distances than droplets. A commonly recommended minimum distance of separation between individuals during the COVID-19 pandemic was two metres. Interventions on populations and communities included closures of schools, workplaces, places of worship and entertainment venues as well as ‘stay-at-home’ orders ('lockdowns') that prevented most people from coming into contact with anyone outside their own homes.

**Test, trace and isolate**

SARS-CoV-2 is transmitted when infected individuals are in close proximity to others. A strategy employed to break the chain of transmission is to identify infectious people ('test'), determine with whom they have come into physical contact ('trace') and encourage or enforce both infected individuals and their contacts to stay at home and avoid physical contact with others until the risk of being infectious has subsided ('isolate').

**Travel restrictions and controls across international borders**

During a pandemic, where an infectious disease is spreading across international borders, restricting the ability of people to move between countries can be used to try to prevent the global movement of the pathogen. Border controls applied during the pandemic varied in stringency and took the form of complete or partial bans targeted at international travellers from particular regions perceived as being at higher risk. Often border controls were accompanied by requirements for international travellers to test and/or quarantine at the border of departure and/or arrival to enable some travel.

**Environmental controls**

Particles carrying infectious material vary in size from droplets that settle on surfaces close to the point of exhalation through to very fine aerosols which can linger in the air and travel further. Certain elements of building design and management can be implemented with the intention of restricting the spread of respiratory pathogens. These include enhancing ventilation systems to replace air carrying infectious aerosols with outside air, and filtering or treating air inside buildings to reduce infectious virus. Screens made of a variety of materials and reduced occupancy limits for rooms or buildings can also be used. Environmental controls also include cleaning of surfaces to remove droplets carrying infectious material and enhanced handwashing.

**Communications**

Effective communication about any of the physical, social or behavioural interventions is essential if people are to understand and be convinced of the reason for their use, as well as being willing to adopt and maintain the practices, and to do so correctly, so as to maximise effectiveness.

From the start of the pandemic, rapidly growing scientific information was deployed continuously to help to control its spread. The genome of the causative virus, SARS-CoV-2, was sequenced from some of the very earliest samples available from infected humans in China. This sequence information enabled the development of precise molecular diagnostic tests that could be used for diagnosis and mass testing of populations, the development of vaccines and continuous monitoring of the evolution of the virus. The development of tests led to the widespread implementation of ‘test, trace and isolate’ interventions early in the pandemic. COVID-19 was the first pandemic in which it was feasible to conduct prophylactic and therapeutic drug trials and to create novel vaccines during the course of the pandemic, saving lives and modifying the outcomes.

However, despite extraordinary scientific capabilities, for most of the first year of the pandemic the only measures available to slow the transmission of the novel virus were NPIs. For those that were infected and seriously ill, there were no specific treatments or preventative measures in the form of drugs or vaccines. The supportive measures of modern medicine, such as oxygen supplementation, pulmonary ventilation and other forms of advanced life support, saved many lives, but did nothing to slow transmission.

## 2. What are NPIs?

The principles behind NPIs are firmly grounded in prior knowledge about the epidemiology and biology of infectious diseases. In essence, the transmission of an infection from one human to another can be prevented if the transmission pathway can be blocked effectively. For an airborne virus such as SARS-CoV-2, effective measures reduce exposure to virus that has been exhaled by infected people (by breathing, talking, coughing or sneezing). Measures that can assist, in theory, include the wearing of face masks, enhanced ventilation and social distancing. Where infectious virus survives on surfaces (furniture, clothes or hands), cleaning regimes including enhanced handwashing can help. Personal protection equipment (PPE), common in healthcare environments (including gloves, visors, gowns and masks) potentially offers protection against exposure.

Early clinical studies of COVID-19 strongly suggested that the primary routes for acquiring infection were likely to be by direct inhalation or exposure of the mucosal surfaces of the nose and mouth to virus suspended in airborne droplets or, as was realised some months into the pandemic, in aerosols. Early evidence of fomites (contaminated surfaces), extensively contaminated with SARS-CoV-2 viral nucleic acid shed from infected people, pointed to the possibility that hand-to-face contact might also transmit the infection.

This view was informed by prior knowledge of the transmission mechanisms of other respiratory viruses, such as influenza, respiratory syncytial virus (RSV) and the coronavirus (now named SARS-CoV-1) that caused the SARS outbreak in several countries around the world in 2003.

## 3. Use of NPIs for infectious disease control

Considering the incomplete knowledge about this new viral infection and prior knowledge, many governments around the world implemented measures similar to those used just over a century earlier during the 1918 influenza pandemic. Some countries in Asia implemented measures based on their more recent experience of outbreaks of SARS and Middle East Respiratory Syndrome (MERS).

NPIs included the wearing of masks and enhanced personal hygiene measures, including enhanced surface cleaning and handwashing. Social distancing was introduced and enforced to variable extents. Social distancing measures included closures of schools and workplaces, as well as entertainment, leisure and sporting venues. These closures were often augmented by stay-at-home orders for all but essential workers. Border controls and closures were put in place in many countries with the aim of reducing the movement of cases across national borders. The precise measures, and the ways they were implemented, varied between countries according to their social and political-economic contexts and prior experiences.

In most of the world, NPIs remained the dominant mechanism for control of the pandemic until well into its second year. The UK was the first country to approve the use of vaccines against SARS-CoV-2, approving three vaccines during December 2020 and January 2021. By July 2021, approximately half of the UK's population had received two doses of vaccine. However, it took until January 2022 for half of the global population to have had two doses – and a year later in January 2023 the global figure had risen to approximately 63% [3].

The challenge for governments around the world facing a pandemic is how to minimise the harms to their populations. The harms of a pandemic are the morbidity and mortality from the viral infection, coupled with the social disruption and harms that follow from the direct and indirect consequences of that morbidity and mortality. The latter can be exceptionally severe if the extent of illness and social response to the illness disrupts the healthcare systems, infrastructure, goods and services on which the health, wellbeing, resilience and security of the population depend.

## 4. Two approaches to assessing the evidence on NPI effectiveness

There are two main approaches to generating and analysing evidence about the effectiveness of any intervention intended to alter health outcomes.

The first and most rigorous approach is to conduct carefully designed **controlled trials**, in which two or more closely matched groups of people are randomised to receive interventions that differ in strictly defined and limited ways. The advantage of this approach is that any changes in health outcome or any side effects of the intervention can be attributed with high confidence to the specific intervention(s).

One potential disadvantage is that typical controlled trials of new interventions include groups of people amounting at most to a few thousand people in each comparison arm, with participants chosen to enter trials chosen on the basis of very strict criteria. Extrapolating the results of such carefully supervised and monitored studies to much larger and more heterogeneous populations ‘in the real world’ is not straightforward.

The intervention may turn out to be less effective in demographically more diverse populations; new and harmful interactions may be discovered when the intervention is provided to people with other conditions or taking other treatments; or rare but important adverse effects may only be discovered when the new intervention reaches a much larger population for the first time.

It is possible to conduct randomised controlled trials (RCTs) in populations, through study designs such as cluster-randomised studies, in which populations rather than individuals are randomised to different interventions.

The second approach is to conduct **observational studies**, ideally with large numbers of individual participants, to evaluate a new intervention by comparing the outcomes with similar observational data, which might be:

— Historical – for example, examining the outcomes in the same population before and after the intervention;
— Geographical – for example, comparing the outcomes in a population receiving the intervention with those in a population not receiving the intervention in a different region of a country or another country;
— Modelled – for example, comparing the outcomes in a population receiving an intervention with modelled data projecting the health outcome in the same population in the absence of the intervention, based on prior observed data about the progression of the condition in that population.

The observational approach has the advantage that an intervention can be evaluated ‘in the real world’ among very large numbers of people. The disadvantage is that there is a risk that the evidence is less reliable, because it may be confounded by other variables between the different groups under observation (e.g. demographic and social differences between the comparison populations, and/or incomplete and non-standardised observational datasets).

In the case of pharmaceutical and biotechnological interventions during the COVID-19 pandemic, controlled clinical trials of drugs and vaccines were conducted in many countries to examine their clinical effectiveness and to identify the side effects of new therapies and vaccines. The data from these trials formed the basis for licensing decisions by regulators. For example, the RECOVERY Trial enrolled more than 47 000 patients into a rigorously designed trial to test the efficacy of anti-inflammatory and anti-viral treatments to see if these could be repurposed for the treatment of the life-threatening consequences of COVID-19 [4]. Similarly, newly created vaccines developed in Europe and the USA against SARS-CoV-2 were tested rigorously and found to be highly effective in reducing severe morbidity and mortality.

In comparison, controlled trials played a relatively small role in the evaluation of NPIs during the pandemic. There were three main reasons for this:

1. The first was that, in the face of significant knowledge gaps and immediate threats to health and life, the need for urgent actions took precedence over designing and implementing complex trials of NPIs in the absence of pre-prepared protocols. At the beginning of 2020, SARS-CoV-2 infection was spreading rapidly across the world. There was early evidence that respiratory spread was very likely to be the dominant route of transmission. NPIs were the only available steps that might slow or stop the spread of infection. These measures were known to be most likely to be effective when applied when infection numbers were still low. So, it was not a dominant consideration for policymakers to undertake prior formal evaluation of NPIs before their large-scale implementation.
2. The second reason was that that NPIs were typically implemented at a national scale, and applied in combinations on the grounds that NPIs would be expected to be complementary in their actions, e.g. masks + handwashing + social distancing + good ventilation. These measures were augmented by local or large-scale ‘lockdowns’ as numbers of cases rose. As soon as accurate diagnostic tests became available at scale, it became feasible to undertake large-scale testing, tracing and isolation of infected individuals and their contacts. These policy approaches to limiting the transmission of SARS-CoV-2 made trials to investigate the efficacy of individual NPIs almost impossible to implement.
3. The third reason was that excellent and rigorous protocols for controlled studies of drugs, vaccines and other biomedical interventions were available ‘off the shelf’. By contrast, similar trials for complex interventions with strong social and behavioural elements are harder to design and implement and historically have been carried out much less frequently. An adequate design for studying the efficacy of NPIs would have needed to include measures of their desired impact in reducing SARS-CoV-2 transmission alongside measures of their potential undesirable impacts on a large variety of personal and societal variables. These ranged from the mental and physical health consequences of social isolation to the consequences of loss of education, jobs and businesses, and broader economic impacts.

This approach to the implementation of NPIs, which largely precluded formal large-scale comparison studies of the effects of different individual NPIs, or of any deliberate comparisons between the effect of packages of NPIs and that of using no NPIs, meant that there were no easy means of evaluating their uptake and effectiveness. There were very few studies of adequate scale to achieve reliable results that compared different types of NPI or that were able to compare, for example, the presence or absence of mask-wearing, or that could measure the effects of different levels of social distancing.

There were however a very large number of observational studies that were performed around the world during the pandemic and it is possible to learn a great deal from well-conducted observational studies performed at large scale. Such observational studies were used to explore the effectiveness of stringent social distancing measures, including stay-at-home orders, and closures of work, school, leisure, entertainment, and sporting facilities. In the case of mask usage, there were comparisons in healthcare settings between masks that provided lesser or greater barrier function. International comparisons were also helpful because some countries took markedly different approaches to the use of NPIs, although demographic and other societal differences mean that these should be interpreted with caution.

## 5. Evidence reviews and national case studies of the effectiveness of NPIs

For the purpose of this report, two approaches were taken to considering the evidence accrued during the pandemic on the effectiveness of NPIs. The first approach was to conduct six evidence reviews [5–10] examining each of the NPIs individually to examine what has been learnt about their effectiveness. Despite all of the caveats about the difficulties of interpreting data from observational studies, clear signals of effectiveness against transmission of SARS-CoV-2 could be discerned from the evidence reviews for several specific measures.

The second approach was to examine observational data on SARS-CoV-2 infections from three of the small number of regions or countries around the world where cases associated with domestic transmission were first identified in early 2020 and were subsequently contained at very low numbers for approximately the first 18 months of the pandemic. These were Hong Kong, New Zealand and South Korea. In each of these, stringent packages of NPIs were implemented and enforced throughout the pandemic until the second half of 2021. By that time there were large waves of the highly transmissible Delta and Omicron variants of SARS-CoV-2, which caused little harm to the vast majority of those that were fully vaccinated, and their national strategies switched to ‘living with the virus’.

The evidence reviews were undertaken with the aim of establishing the quality and strength of the deductive evidence about the effectiveness of individual NPIs. They were conducted according to a rigorous well-established methodology, which was originally developed to bring together evidence from well-designed clinical trials. When this methodology was applied to observational studies of NPIs it highlighted the inevitable limitations of these studies. Firstly, because interventions were almost invariably implemented in combinations, it was extremely hard to distinguish and measure the effects of any single intervention independently of the others. Secondly, many studies used routinely collected data sets, which were not designed with *post hoc* evaluation in mind. Thirdly, comparison groups were not always included and when available, they were rarely well matched. These and other limitations are classified in such evidence reviews as causing potential biases in the outcomes of individual studies. The word ‘bias’, when used in this way, does not have the same meaning as it does when used in common parlance. Specifically, it does not imply that the researchers were biased or partial in seeking a particular outcome for their research, but instead that there were inherent characteristics in the study design that could reduce the reliability of the conclusions of the research. Such biases could result in either overestimation or underestimation of a measured effect.

The evidence reviews focused on the effectiveness of NPIs in relation to the transmission of SARS-CoV-2 infection (box 2). They did not attempt to explore indirect, social or economic impacts. Nor did they attempt to explore the effects of social context and implementation style on effectiveness; these matters would have required complementary studies using different methods, including qualitative analysis.

Box 2. **What has been learnt about NPI effectiveness?**

**Masks and face coverings**

The weight of evidence from all studies suggests that wearing masks, particularly higher quality masks (respirators), supported by mask mandates, generally reduced the transmission of SARS-CoV-2 infection. Studies consistently, though not universally, reported that mask wearing and mask mandates were an effective approach to reduce infection. There is also evidence, mainly from studies in healthcare settings, that higher-quality ‘respirator’ masks (such as N95 masks) were more effective than surgical-type masks. The evidence suggested that masks with greater barrier function were more effective than those with lower barrier function; and mask wearing in the context of a mandate to wear masks was more effective than mask wearing in the context of voluntary behaviour.

**Social distancing and ‘lockdowns’**

Most effective of all the NPIs were the social distancing measures. Stay-at-home orders, physical distancing, and restrictions on gathering size were repeatedly found to be associated with significant reduction in SARS-CoV-2 transmission, with more stringent measures having greater effects. Early in the pandemic certain sub-populations, such as the elderly, were found to be particularly vulnerable to severe disease and death resulting from SARS-CoV-2 infection. Social distancing measures aimed specifically at protecting the elderly, such as restrictions on visitors and ‘cohorting’ staff with residents in care homes (separating residents into groups, each cared for by a specific group of staff), were frequently associated with reduced transmission and reduced outbreaks within care homes. Regarding school closures and other school-based measures, the evidence suggests that they were associated with reduced COVID-19 incidence within schools and the community. However, the effectiveness of these measures was varied (compared to community-wide measures such as stay-at-home orders), time-dependent, and often contingent on the adherence to the measures implemented and the targeted age group of school children.

**Test, trace and isolate**

Test, trace and isolate approaches were used as a key intervention in many countries, especially those pursuing zero-COVID policies. Studies from several countries that implemented high levels of contact tracing with isolation of infected individuals and their contacts found reductions in COVID-19 deaths. Strong evidence was also found for the effectiveness of contact tracing apps. For example, a trial of the UK's app (alongside communications and manual tracing interventions) on the Isle of Wight was associated with a substantial reduction in transmission [11].

**Travel restrictions and controls across international borders**

Observational evidence from national case studies, including New Zealand, showed that comprehensive border control policies could reduce but not eliminate the number of infected travellers or their contacts at the borders entering the country. However, despite most countries introducing travel restrictions during the COVID-19 pandemic, few studies have been published so far examining the effectiveness of these measures when implemented alone. Based on the available evidence, symptomatic or exposure-based screening, including temperature screening before travel, was found to have had no meaningful effect on reducing importation or transmission. Targeted travel restrictions including banning entry early in the pandemic from specific countries probably had a moderate effect on transmission but quickly became less effective once the number of cases rose, whereas quarantine at entry borders was found to have the highest levels of effectiveness.

**Environmental controls**

The review found evidence that enhanced ventilation, air treatment to remove infectious virus and reduced room occupancy did reduce transmission within particular settings. However, these measures were typically applied in combination with other NPIs, so accurately and individually quantifying their effectiveness was not possible. Many were observational studies conducted retrospectively rather than planned prospectively. As a consequence the studies were unable to control fully for possible confounding factors. It is also the case that the effectiveness was only judged within the setting in which the control was applied, and not at the wider population level. There was insufficient evidence to judge the effectiveness of enhanced surface cleaning or the use of barriers. These are important gaps where laboratory studies could help provide insight.

**Impact of communication in the UK on uptake of NPIs**

Communications in this review were considered specifically in the UK context because political, social and cultural differences make it extremely hard to extrapolate findings about the effectiveness of communications from one country to another. The limited evidence confirmed that communication was sufficiently effective to ensure high adherence to NPIs, although also identifying the characteristics that led to non or less rigorous adherence. Trust and confidence in those communicating was important as was the clarity and consistency of the messaging and the opportunity for personal control. The limited evidence suggests that social media communications are less likely to be associated with higher adherence than those via the traditional media.

## 6. Three country experiences with NPIs to control viral transmission

There are important lessons to be learnt from how different nations implemented NPIs to control the transmission and spread of SARS-CoV-2. The implementation of NPIs differed between and within different countries by time, region, and stringency. There were prominent differences in the timing and intensity of test and tracing, social distancing and ‘lockdown’ measures. Asian countries that had more recently experienced SARS and other emerging infectious diseases, including MERS and avian influenza, such as China, Hong Kong, Taiwan, Singapore, South Korea and Vietnam, used that experience to take a strategic approach aimed at reducing transmission and thereby slowing the spread of infection as quickly as possible. These countries implemented early stringent NPIs, followed by Australia and New Zealand [12].

Three case studies from Hong Kong, New Zealand and South Korea (summarised in box 3) are used to illustrate these lessons. Over the course of the pandemic these were among a small number of locations worldwide that maintained low rates of transmission over a prolonged period.

Box 3. **Summary of case studies of countries that maintained low levels of transmission for a prolonged period of time.**

**Hong Kong special administrative region**

Hong Kong suffered some of the more severe effects of the SARS outbreak in 2003, experiencing almost a quarter of the 8098 cases worldwide, with 302 deaths. This precipitated significant public investment in health infrastructure and diagnostic testing capacity. Strict policies were put in place during the COVID-19 pandemic that required those who tested positive to isolate for 21 days and those with whom they had been in contact to isolate for 14 days. Quarantine at borders for international travellers was similarly strict. It was estimated that only 27% of all cases that occurred in Hong Kong were confirmed by laboratory test, meaning that Hong Kong's containment of the pandemic cannot be attributed to these policies alone. Further measures included minimum distancing, curfews on restaurant opening times, bans on large events, requirements to work from home and school closures. Mask wearing was also mandated in all public settings with high compliance from the population. Vaccines were used to immunise approximately 60% of the population by the end of 2021. Uptake was lower in older adults. When the more transmissible Omicron variant arrived and rapidly spread, more than 10 000 deaths occurred largely in vulnerable elderly unvaccinated people.

**New Zealand**

New Zealand is a geographically isolated island group with a small population and hence is atypical. However, it is a useful example of how a country developed and implemented a national strategy for use of NPIs to enable the prolonged control and near elimination of SARS-CoV-2 infection. This strategy was built around stringent border controls, including tightly restricted entry criteria, with pre-departure and post-arrival testing of travellers; 14-day quarantine (initially by self-isolation, subsequently by supervised hotel-managed isolation and quarantine); strict test, trace and isolate measures; and local or national ‘lockdowns’ when domestic transmission was detected or at high risk of occurrence.

This approach controlled the initial outbreak of COVID-19 in New Zealand, where the first recorded case was on 28 February 2020. By 8 June 2020, all domestic NPIs had been lifted and a total of 1504 cases and 22 deaths had been recorded. New Zealand remained mostly transmission-free until late 2021, despite regular positive tests among quarantined international arrivals.

The more transmissible Delta variant of SARS-CoV-2 was first detected in August 2021. By this stage the population of New Zealand was highly vaccinated and facing an increasing number of daily cases and the prospect of an extended ‘lockdown’, the government declared the end of the elimination strategy on 4 October 2021.

Whilst local NPIs were eased at this time, strict border controls remained in place. In mid-December, the highly transmissible Omicron variant was first detected in entering travellers. Community transmission was not identified until 23 January 2022, and this was followed by a large wave of Omicron infections across New Zealand.

**South Korea**

South Korea had experienced an outbreak of MERS in 2015 in which there were 186 cases and 38 deaths. This experience had prompted significant policy reform for pandemic preparedness. Testing infrastructure was well established and ready to be rolled out nationwide in drive-through testing facilities. Testing provided effective estimates of caseload in the country and was coupled with innovative use of technology to great effect. Global Positioning System (GPS) data from mobile phones were used to monitor movements of citizens who were alerted if they had been near a confirmed COVID-19 case and instructed to isolate. Arrivals from other countries were quarantined for 14 days at the border and those from Hubei in China were banned outright.

Citizen compliance with policies designed to mitigate transmission was also demonstrably higher than it had been during the MERS outbreak, suggesting that the population was more conscious of the risks around an emerging respiratory disease. The early adoption of these packages of NPIs contained the pandemic effectively and meant that an early ‘lockdown’ was avoided.

These national and regional case studies show that it was possible, in certain contexts, to control transmission of SARS-CoV-2 for over a year by implementing early, stringent border controls accompanied by other strict NPIs to prevent and control domestic transmission. They also demonstrate that the effectiveness of NPIs varied inversely in relation to the transmissibility of the infection. As the pandemic progressed, the evolution of increasingly transmissible variants, particularly Omicron, became harder and harder to control using even the most stringent application of NPIs. However, by this point in the pandemic, effective vaccines were becoming widely available and countries pursuing ‘zero-COVID’ strategies switched to policies of high vaccine coverage and ‘living with COVID’. This adjustment was seen in all three of the country case studies, despite early success in containing the pandemic.

However, the results reported in the three national and regional case studies cannot simply be replicated in other countries and regions. The national and regional contexts for NPIs varied significantly around the world, according to geographical, political, demographic, socio-economic and regulatory factors. The nature of the national implementation of NPIs and their resulting effectiveness can only be understood in the context of a series of other extremely important interacting factors.

Cross-country comparisons of the effectiveness of NPIs are affected by multiple factors, most notably differences in demographic factors, healthcare systems, levels of economic prosperity, degrees of trust between citizens and public authorities, and testing and reporting of cases of COVID-19. Different countries or regions were differentially affected by COVID-19 with particular impacts on those with older populations; [13] higher levels of obesity [14]; greater incidence of chronic non-communicable diseases such as diabetes and cardiovascular disease; larger concentrations of lower-income and larger households; and higher population densities [15].

Countries also differed in their categorisation of COVID-19 deaths. For instance, Belgium included all deaths where COVID-19 was suspected to contribute, resulting in higher reported death rates early in the pandemic [16], while others included only deaths in hospitals [17]. There were also stark differences in the availability of testing and thereby the numbers of reported cases.

## 7. Conclusion

There is clear evidence from studies conducted during the pandemic that the stringent implementation of packages of NPIs was effective in some countries in reducing the transmission of COVID-19. There is also evidence for the effectiveness of individual NPIs, although, especially as the pandemic progressed and the virus became more transmissible, NPIs became less effective in controlling the transmission of SARS-CoV-2.

A common denominator of the evidence from the studies of individual NPIs and from the national case studies is that NPIs were, in general, more effective when the case numbers and the associated transmission intensity of SARS-CoV-2 were lower. This is because the size of the exposure, and therefore the risk of infection, of uninfected, non-immune people to viral infection is proportional to the number of cases in the community. Similarly, the stringency of the application of individual NPIs and groups of NPIs was important, so there was evidence that respirator masks were more effective than surgical masks and that two weeks of quarantine were more effective than shorter periods.

## 8. Lessons for the future

There are important lessons for the future. For policymakers and their professional advisers, there is a need to learn from national and international experience of the implementation of NPIs during the COVID-19 pandemic, and to understand in detail the differing national contexts and ways in which NPIs were implemented. National context was an important influence on the outcome of the COVID-19 pandemic.

For researchers and their funders, there is a lesson that observational studies can be facilitated if national and international collaborations can be established in advance of a future pandemic, with standardised protocols for data collection. While RCTs should not be discounted, it is highly likely that most information in a future pandemic will continue to be observational. It should be possible to exploit more effectively, for the purposes of evaluation, the consequences of differences in the implementation of NPIs within and between countries and this would be much easier to achieve if protocols could be prepared in advance. So for the future, it is important to design protocols for observational research that can disaggregate the effects of NPIs by social groups and other demographic factors within countries.

Future assessments should also consider the costs as well as the benefits of NPIs, in terms of their impacts on livelihoods, economies, education, social cohesion, physical and mental wellbeing, and potentially other aspects. Drug regulators are able to make recommendations on the use of drugs based upon evidence of their effects and side effects. Similarly, policymakers will be able to make the best policy decisions on NPIs, which are in the main complex social interventions, if they have access to better evidence regarding their broader health and societal impacts. They could consider these alongside their effects on reducing the transmission of the infectious agent. The provision of such evidence will require pre-planned protocols, and in some cases prior research, to collect a wide variety of relevant health and social data systematically and, alongside this, an embedded system of expert research advice to assist policymakers in making extremely difficult policy decisions in the face of a severe pandemic.

The evidence assembled for the development of this report shows that, in the context of COVID-19 that was caused by a virus dominantly transmitted by a respiratory route, controlling the transmission of the virus required a clear plan for the stringent application of combinations of NPIs.

One of the most important lessons from this pandemic is that it proved possible to influence the outcome of the COVID-19 pandemic by means of the rapid development, evaluation and implementation at scale of specific treatments and vaccines. The effective application of NPIs ‘buys time’ to allow the development, evaluation and manufacturing of such therapies and vaccines at scale. So there is every reason to think that the application of combinations of NPIs will be important in future pandemics, particularly at early stages with novel pathogens when there are knowledge gaps and when therapeutics and vaccines are not yet available.

## Data Availability

This article has no additional data.

## References

[1] The Royal Society. 2023 COVID-19: examining the effectiveness of non-pharmaceutical interventions. London: The Royal Society.

[2] How did we get here: what are droplets and aerosols and how far do they go? A historical perspective on the transmission of respiratory infectious diseases. Interface Focus. 10.1098/rsfs.2021.0049 PMC8504878 34956601

[3] Mathieu E *et al*. 2020 Data from: Coronavirus Pandemic (COVID-19). Our World in Data. See https://ourworldindata.org/covid-vaccinations (accessed on 5 July 2023).

[4] RECOVERY. See https://www.recoverytrial.net/ (accessed 5 July 2023).

[5] Boulos L *et al*. 2023 Effectiveness of face masks for reducing transmission of SARS-CoV-2:a rapid systematic review. Phil. Trans. R. Soc. A **381**, 20230133. (10.1098/rsta.2023.0133) 37611625 PMC10446908

[6] Murphy C *et al*. 2023 Effectiveness of social distancing measures and lockdowns for reducing transmission of COVID-19 in non-healthcare, community-based settings. Phil. Trans. R.Soc. A **381**, 20230132. (10.1098/rsta.2023.0132) 37611629 PMC10446910

[7] Littlecott H, Herd C, O'Rourke J, Chaparro LT, Keeling M, James Rubin G, Fearon E. 2023 Effectiveness of testing, contact tracing and isolation interventions among the general population on reducing transmission of SARS-CoV-2: a systematic review. Phil. Trans. R. Soc. A **381**, 20230131. (10.1098/rsta.2023.0131) 37611628 PMC10446909

[8] Grépin KA, Aston J, Burns J. 2023 Effectiveness of international border control measures during the COVID-19 pandemic: a narrative synthesis of published systematic reviews. Phil. Trans. R. Soc. A **381**, 20230134. (10.1098/rsta.2023.0134) 37611627 PMC10446907

[9] Madhusudanan A, Iddon C, Cevik M, Naismith JH, Fitzgerald S. 2023 Non-pharmaceutical interventions for COVID-19: a systematic review on environmental control measures. Phil. Trans. R. Soc. A **381**, 20230130. (10.1098/rsta.2023.0130) 37611631 PMC10446906

[10] Williams SN, Dienes K, Jaheed J, Wardman JK, Petts J. 2023 Effectiveness of communications in enhancing adherence to public health behavioural interventions: a COVID-19 evidence review. Phil. Trans. R. Soc. A **381**, 20230129. (10.1098/rsta.2023.0129) 37611630 PMC10446905

[11] Kendall M et al. 2020 Epidemiological changes on the Isle of Wight after the launch of the NHS Test and Trace programme: a preliminary analysis. The Lancet Digital Health **2**, e658-e666. (10.1016/S2589-7500(20)30241-7) 33078140 PMC7556784

[12] Pearce N, Lawlor DA, Brickley EB. 2020 Comparisons between countries are essential for the control of COVID-19. Int. J. Epidemiol. **49**, 1059-1062. (10.1093/ije/dyaa108) 32601669 PMC7337754

[13] Dowd JB, Andriano L, Brazel DM, Rotondi V, Block P, Ding X, Liu Y, Mills MC. 2020 Demographic science aids in understanding the spread and fatality rates of COVID-19. Proc. Natl Acad. Sci. USA **117**, 9696-9698. (10.1073/pnas.2004911117) 32300018 PMC7211934

[14] Chaudhry R, Dranitsaris G, Mubashir T, Bartoszko J, Riazi S. 2020 A country level analysis measuring the impact of government actions, country preparedness and socioeconomic factors on COVID-19 mortality and related health outcomes. eClinicalMedicine **25**, 100464. (10.1016/j.eclinm.2020.100464) 32838237 PMC7372278

[15] Banholzer N, Feuerriegel S, Vach W. 2022 Estimating and explaining cross-country variation in the effectiveness of non-pharmaceutical interventions during COVID-19. Sci. Rep. **12**, 7526. (10.1038/s41598-022-11362-x) 35534516 PMC9085796

[16] Molenberghs G *et al*. 2020 COVID-19 mortality, excess mortality, deaths per million and infection fatality ratio, Belgium, 9 March 2020 to 28 June 2020. Euro Surveill. **27**, 2002060. (10.2807/1560-7917.ES.2022.27.7.2002060) PMC8855510 35177167

[17] Stokes AC, Lundberg DJ, Bor J, Bibbins-Domingo K. 2021 Excess Deaths During the COVID-19 Pandemic: Implications for US Death Investigation Systems. Am. J. Public Health. **111**, S53-S54. (10.2105/AJPH.2021.306331) 34314220 PMC8495654

